# Supplementary material for: Crystal plasticity as an indicator of the viscous-brittle transition in magmas
Source: Nat Commun. 2017 Dec 4;8:1926. doi: 10.1038/s41467-017-01931-4 (PMC5715024; doi:10.1038/s41467-017-01931-4)
Supplement: Supplementary file 1 — Supplementary Information [file 41467_2017_1931_MOESM1_ESM.pdf]

Supplementary Table 1: Sample locations and details for the blocks collected from Volcán de Colima (Mexico).

Sample name	Location	Comment
COLB2	N 19° 29' 14,8" /W 103° 35' 34,8"	1975-6 lava flow
COLLAH4	N 19° 27' 08,3" /W 103° 42' 46,3"	River bed, remobilised block

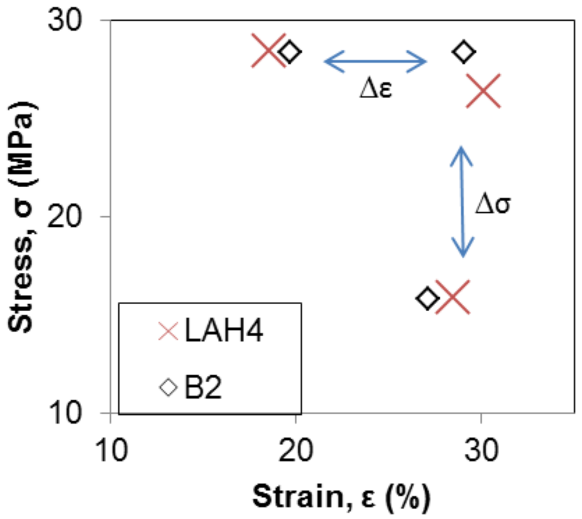

Supplementary Figure 1: A plot to show the stress and strain conditions of the experiments conducted on the two andesitic lavas from Volcán de Colima; COLB2 and COLLAH4.

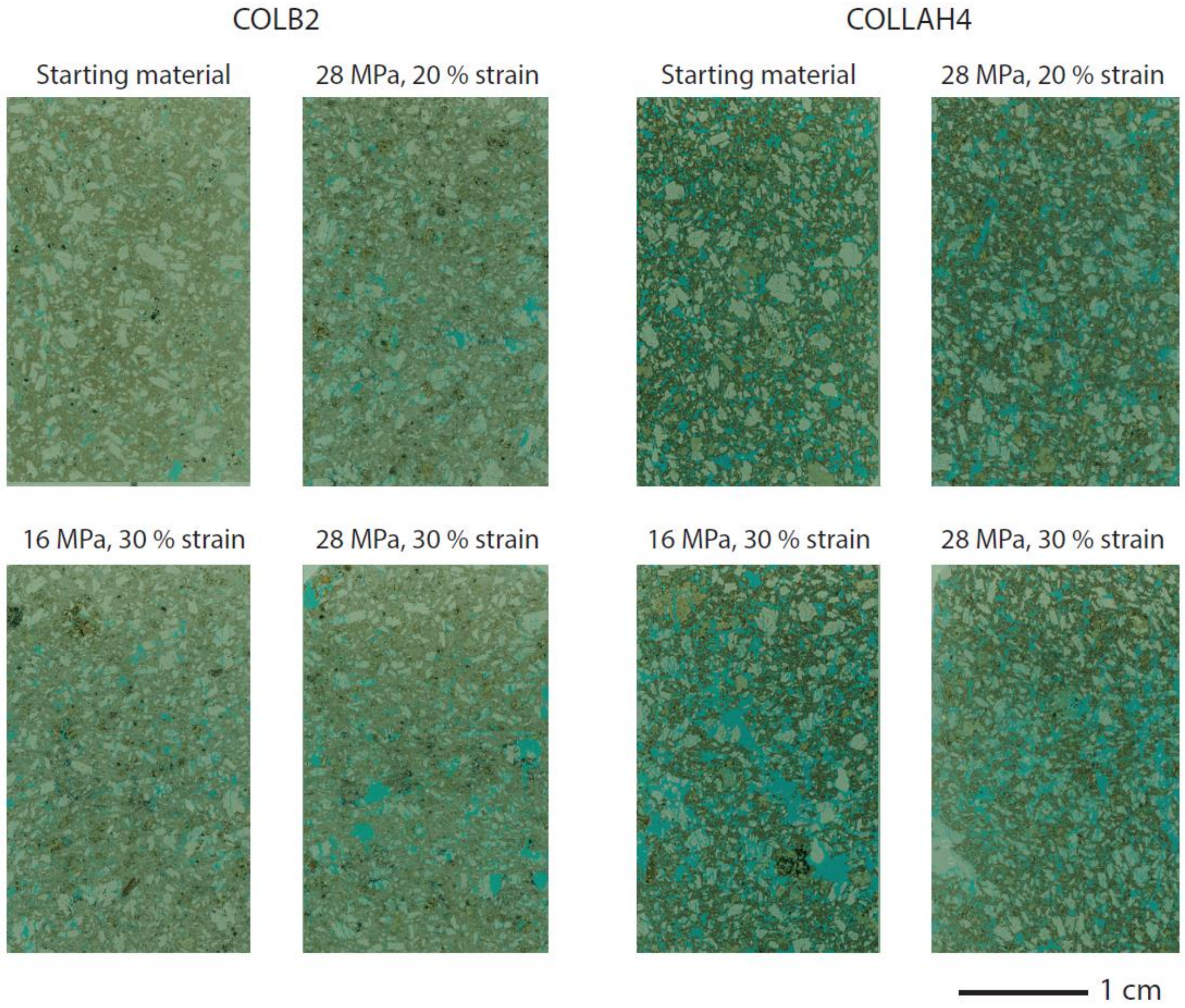

Supplementary Figure 2: Thin section scans from the starting materials COLB2 and COLLAH4 and from the central portion (across the diameter, parallel to the length) of the six experimentally deformed cores. Images give an overview of the distribution of cracking throughout the samples when the principal stress is applied in the vertical direction. An increasing amount of damage with increasing stress and /or strain is evident in both samples. At low stresses and strains fractures are largely restricted to phenocrysts, and individual fractures are predominantly parallel to applied stress in both COLB2 and COLLAH4. At higher stresses and strains fractures propagate through the groundmass as well and fractures coalesce to form pervasive fractures and damage zones, which form at approximately 45° to applied load. Microstructural analyses for this study are carried out in the central portion of all samples, which is not influenced by friction or pressure shadows produced by contact with the upper and lower pistons.

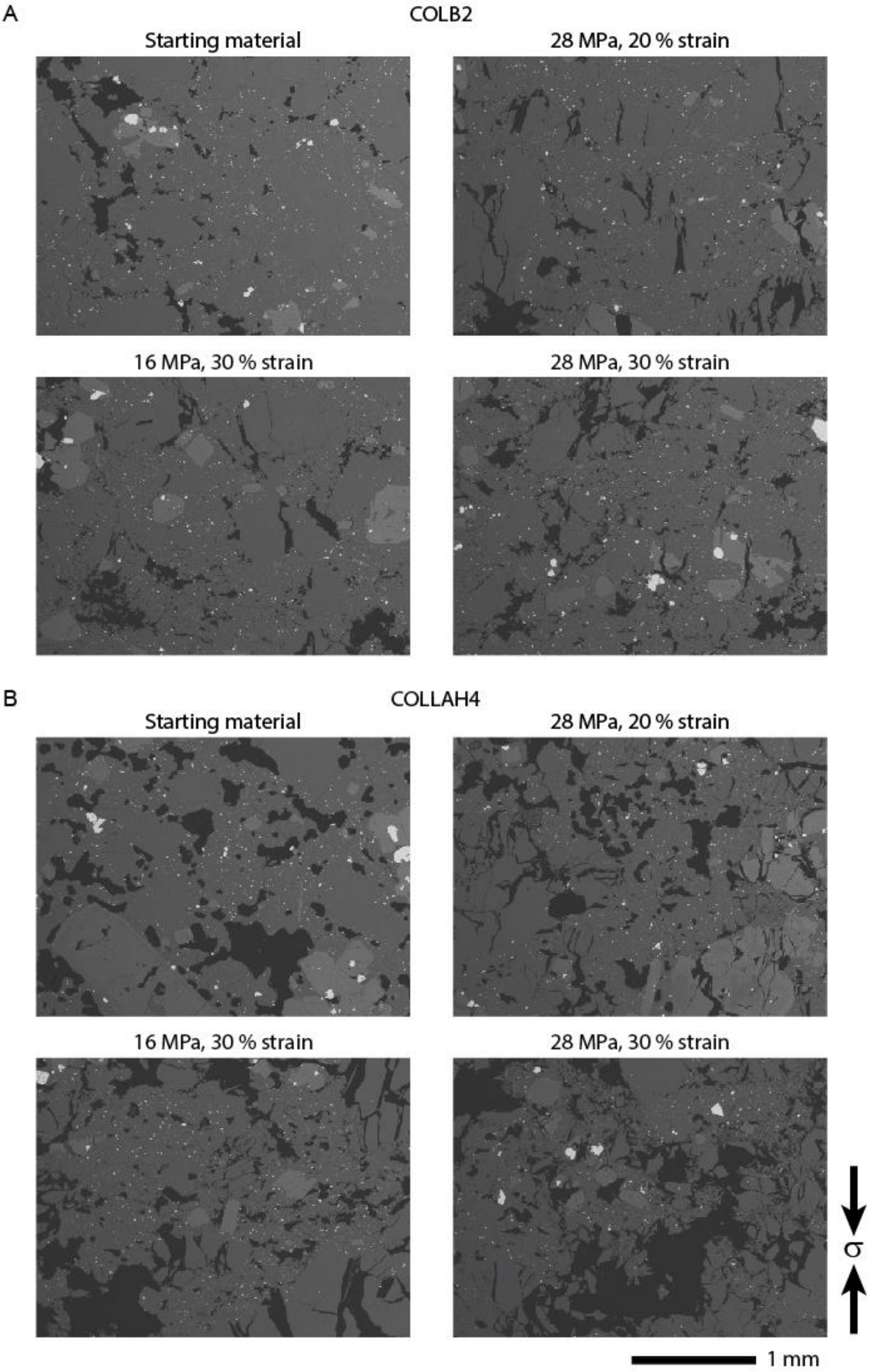

Supplementary Figure 3: Backscatter electron (BSE) images of the deformed samples, imaged using a Philips XL 30 scanning electron microscope (SEM). For deformed samples compression direction is vertical in the current view. In A, the denser COLB2 starting material has relatively few pores, and those present are small and intermittently clustered (dark areas). In B, the more porous COLLAH4 sample has more, larger, vesicle-shaped pores. In both samples, applying stress / strain resulted in fracturing, first in the crystals, where fractures formed parallel to the compression direction, and then as stress or strain increased, the fractures coalesced through the interstitial glass, and grain crushing became dominant (see also Supplementary Figures 4-5, and Kendrick et al.[1] for a discussion).

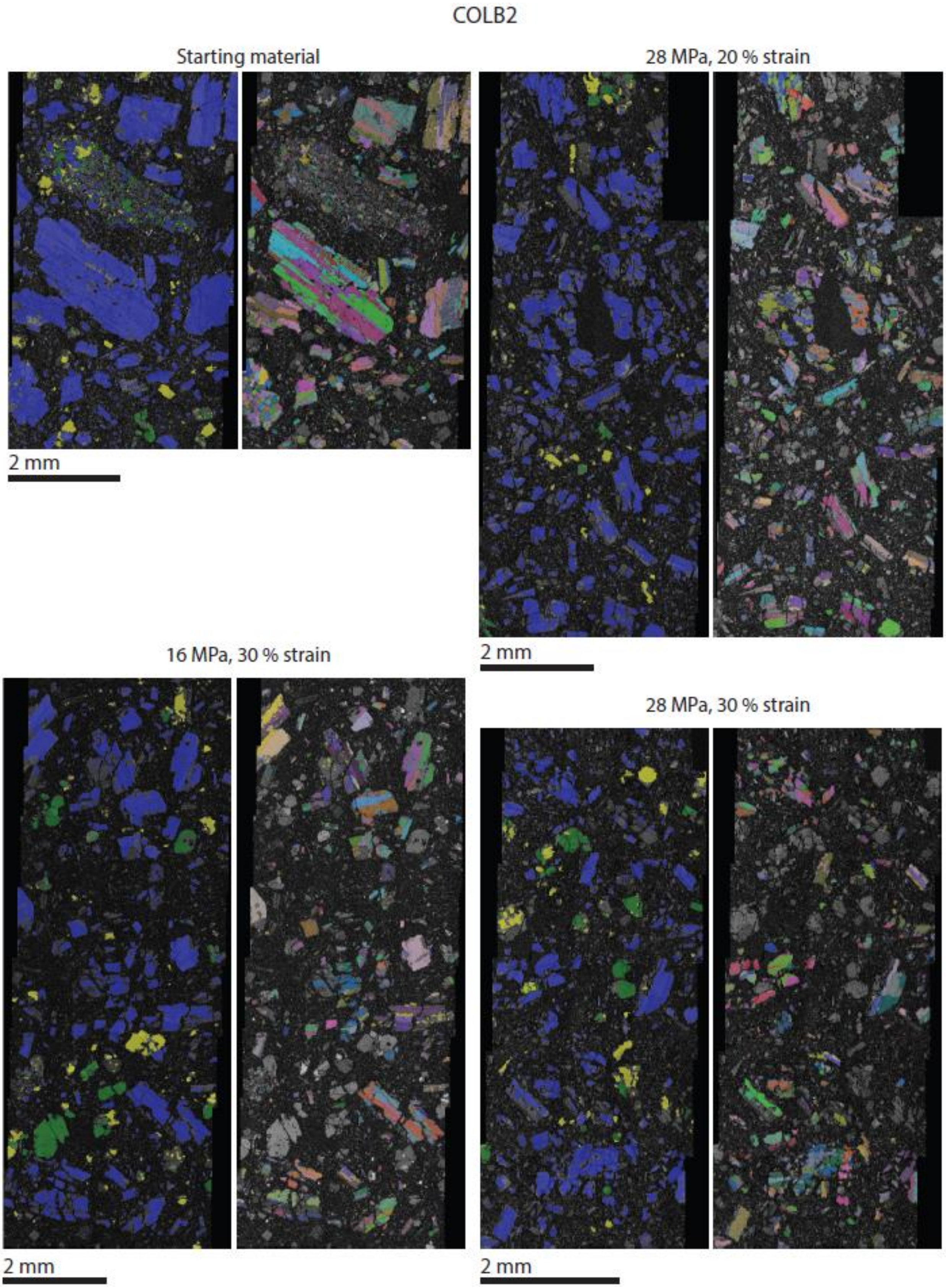

Supplementary Figure 4: EBSD pattern quality (band contrast) maps of "as-collected" starting material and deformed COLB2 samples overlain by indexed points, measured with 15 µm step size (see Methods for other parameters). On the left side of each pair of images, crystalline phases are identified; blue (plagioclase), green (diopside) and yellow (hypersthene). On the right, all Euler colours for the plagioclase only are superimposed onto the band contrast image, here x is right, y is vertical. These images show the net grain size reduction due to phenocryst fracturing with increasing stress/ strain, with the principal stress direction vertical in all images (the starting material is cut and imaged in the same orientation).

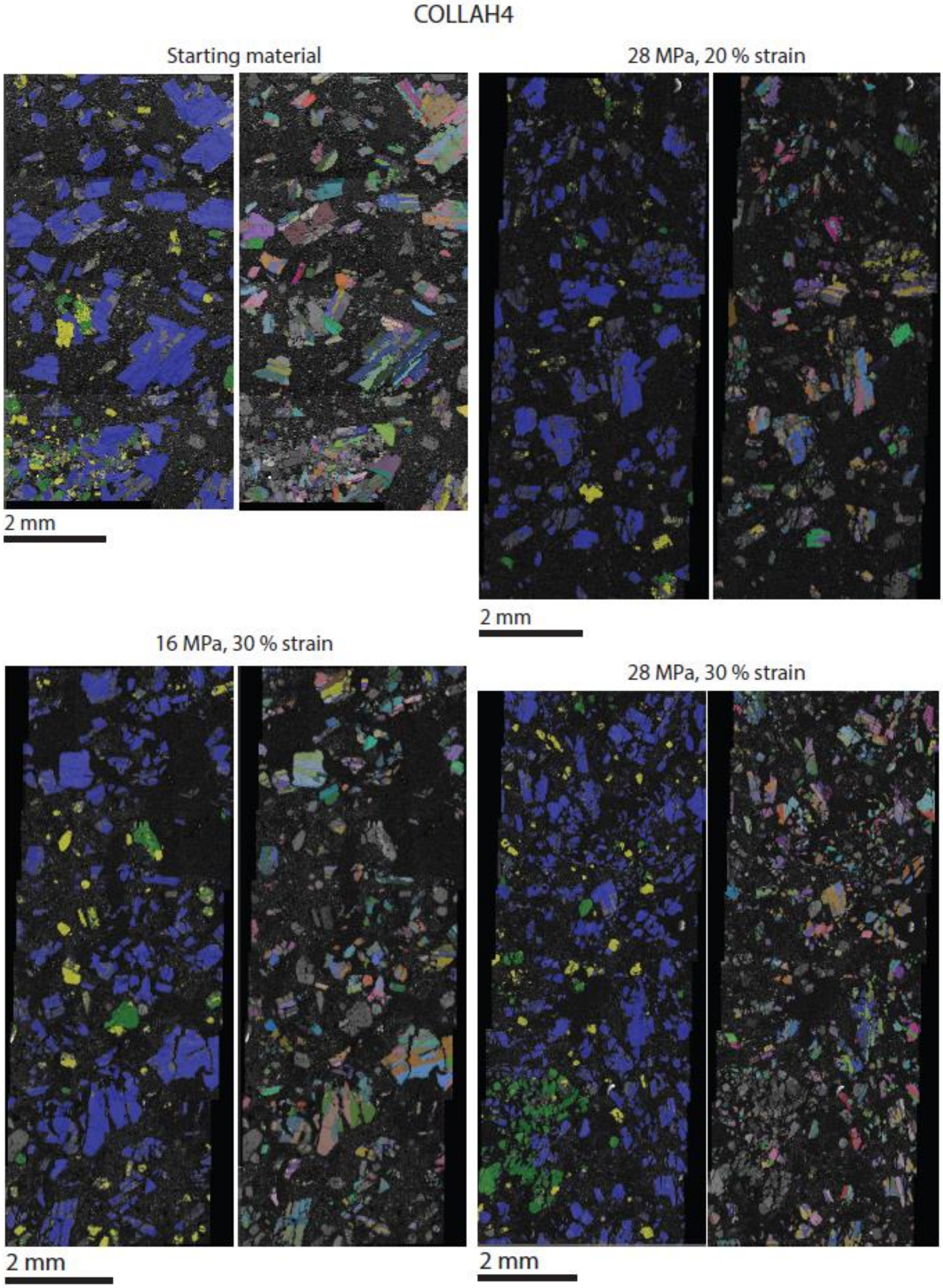

Supplementary Figure 5: EBSD pattern quality (band contrast) maps of “as-collected” starting material and deformed COLLAH4 samples overlain by indexed points, measured with 15 µm step size (see Methods for other parameters). On the left side of each pair of images, crystalline phases are identified; blue (plagioclase), green (diopside) and yellow (hypersthene). On the right, all Euler colours for the plagioclase only are superimposed onto the band contrast image, here x is right, y is vertical. These images show the net grain size reduction due to phenocryst fracturing with increasing stress/ strain, with the principal stress direction vertical in all images (the starting material is cut and imaged in the same orientation).

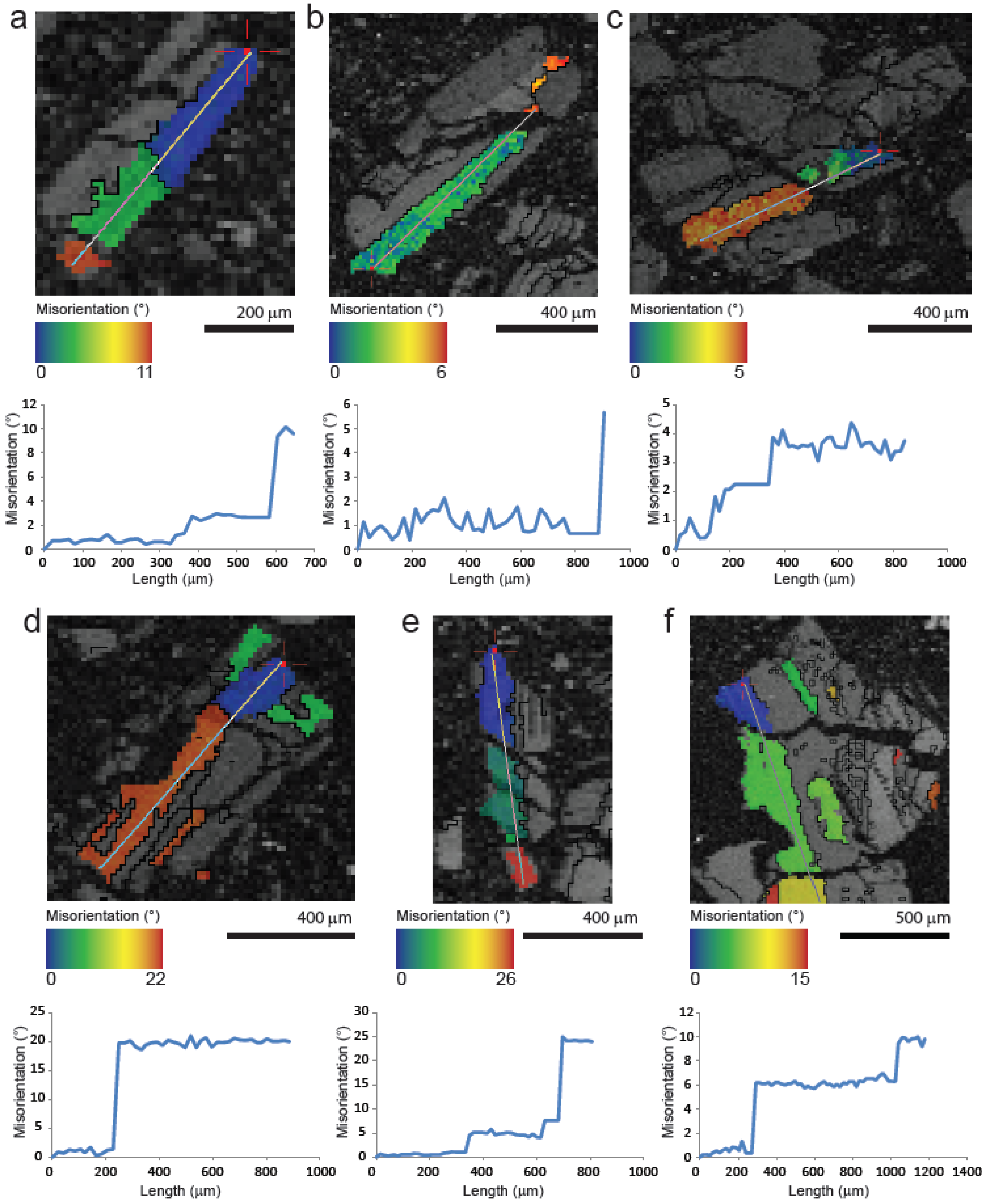

Supplementary Figure 6: A number of phenocrysts evidencing brittle fractures in the as-collected dome lavas (a and b) and the experimentally deformed samples (c to f), with the principal stress direction horizontal in all images, here x is right, y is vertical. The texture component maps indicate the distortion of the crystal from the red cross, and the misorientation profiles show the transects marked on the crystals (from the red cross at length 0 at the blue end of the microlite). Unlike the microlites, the dominant deformation mechanism in the phenocrysts is brittle, and misorientation comprises sharp jumps across fractures, while intact portions of the crystals show no systematic distortion (plasticity).

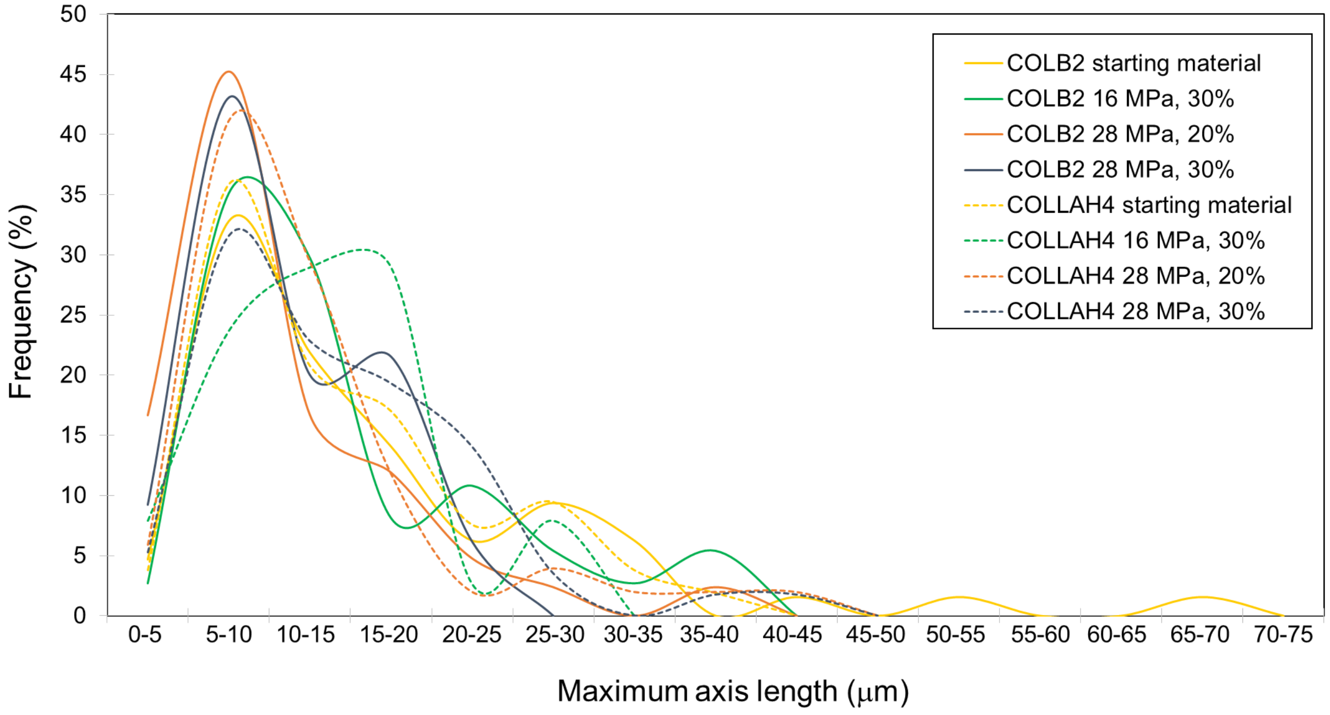

Supplementary Figure 7: Grain size distributions in terms of long-axis length for natural and deformed sample COLB2 and sample COLLAH4 from all microlites identified in the maps in Supplementary Figures 8-9, details in Supplementary Data 2-9

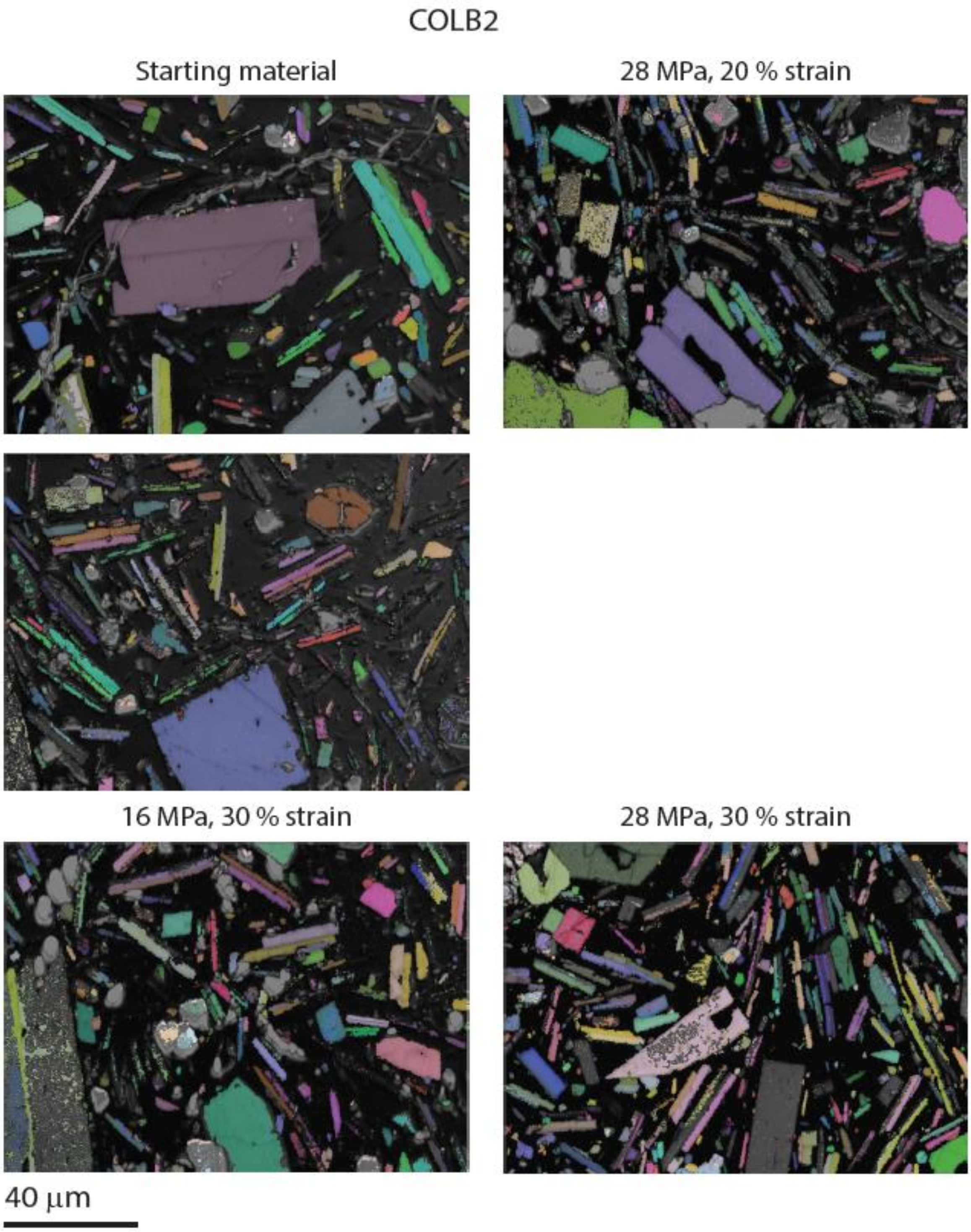

Supplementary Figure 8: EBSD pattern quality (band contrast) maps of "as-collected" starting material and deformed COLB2 samples overlain by indexed points, measured with 0.2 μm step size (see Methods for other parameters). All Euler colours are superimposed onto the band contrast image of the microlites, and where less than 40 microlites are present in the first map, a second map is given. These maps are used for the microstructural analysis presented in the paper, and the distortion data for all crystals is given in Supplementary Data 2-5, while broken crystals are in Supplementary Data 10. The principal stress direction is vertical in all images (the starting material is cut and imaged in the same orientation), while x is right, y is vertical.

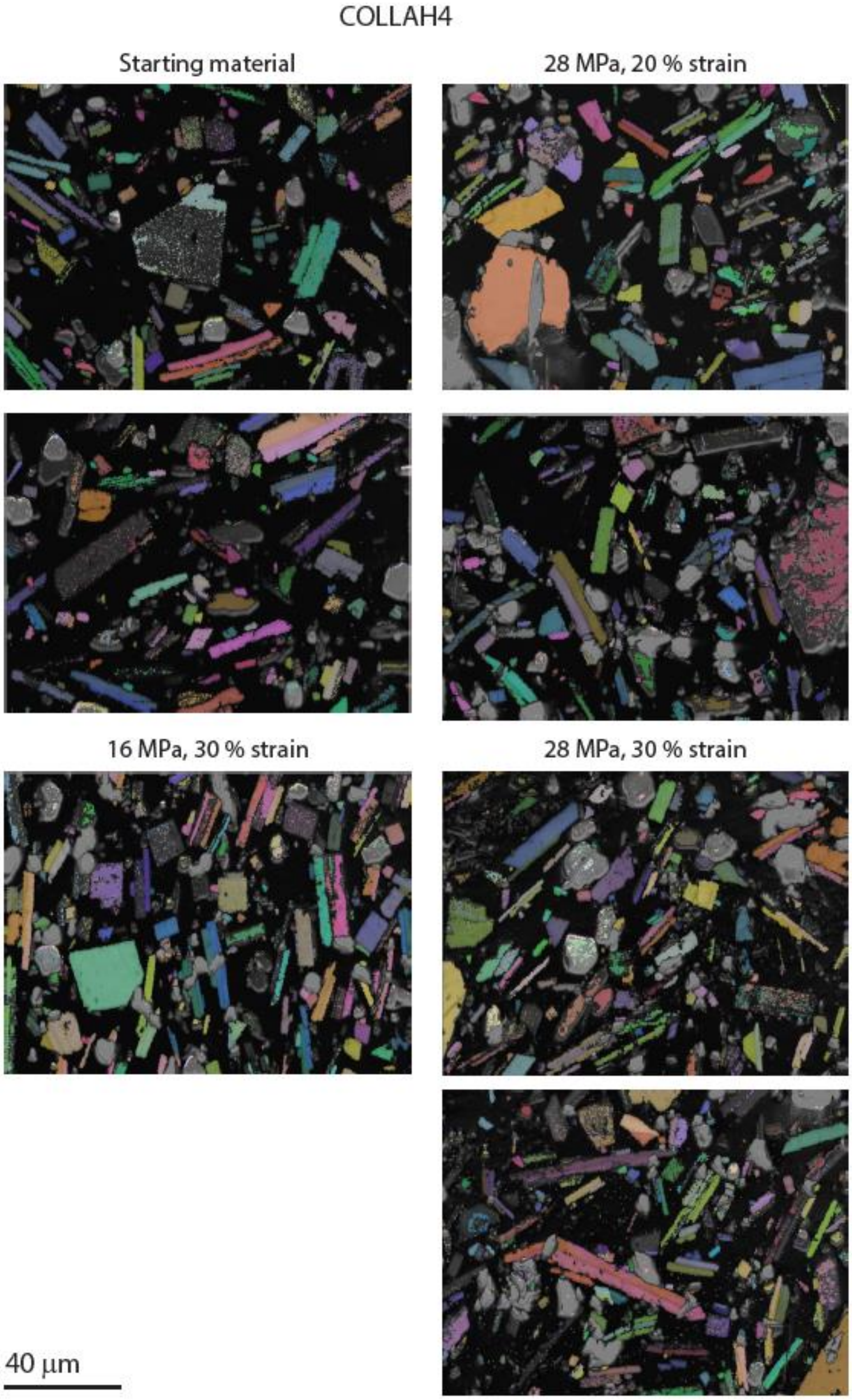

Supplementary Figure 9: EBSD pattern quality (band contrast) maps of "as-collected" starting material and deformed COLLAH4 samples overlain by indexed points, measured with 0.2 μm step size (see Methods for other parameters). All Euler colours are superimposed onto the band contrast image of the microlites, and where less than 40 microlites are present in the first map, a second map is given. These maps are used for the microstructural analysis presented in the paper, and the distortion data for all crystals is given in Supplementary Data 6-9, while broken crystals are in Supplementary Data 11. The principal stress direction is vertical in all images (the starting material is cut and imaged in the same orientation), while x is right, y is vertical.

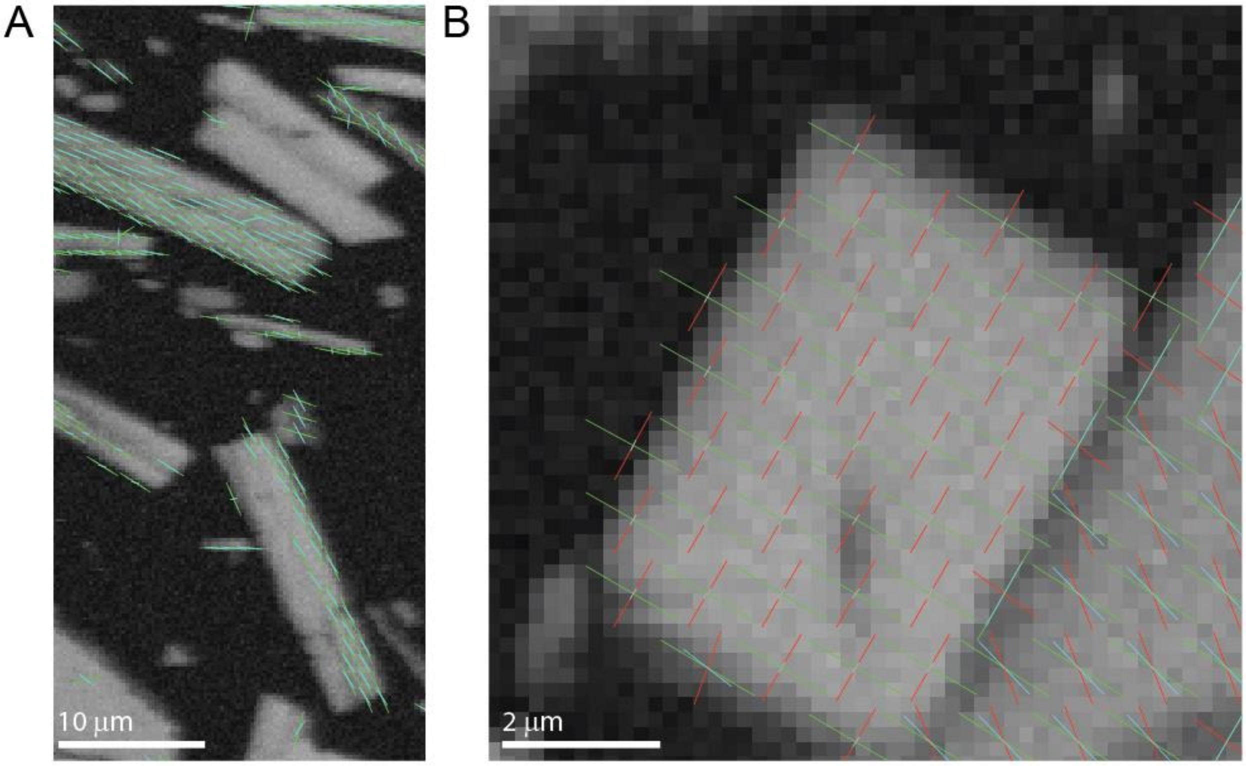

Supplementary Figure 10: Crystallographic information superimposed on band contrast maps with [100], the *a* axis, in blue, trace of (010) plane in green and trace of (001) plane in red. A) Side view of laths with [100], the *a* axis, and trace of (010) subparallel to length and B) shows end-on lath with faces parallel to (010) and (001) and hence their intersection (long direction) is parallel to the *a* axis, in this case almost perpendicular to the map.

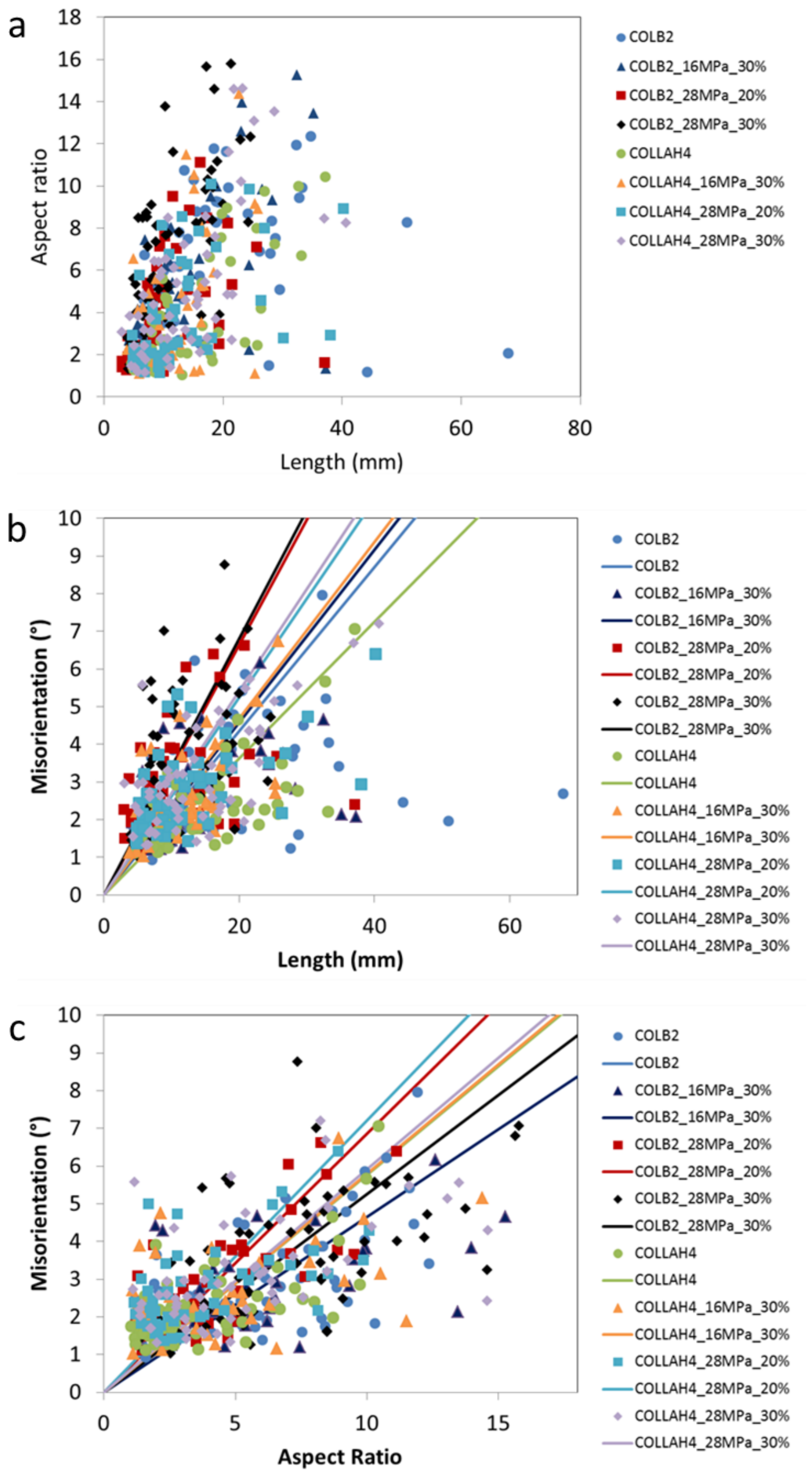

Supplementary Figure 11: A comparison of the relationship of length, aspect ratio and distortion for microlites: (a) Length versus aspect ratio by sample set showing a subtle positive correlation suggesting that as microlites grew in the magma they also became more elongate, we also see that microlites in COLB2 tend to have higher aspect ratios for a given length, and that aspect ratio slightly decreases with deformation, which results from crystal fracturing. Maximum misorientation angle (amount of distortion) in a microlite is shown (b) as a function of length and (c) aspect ratio. The comparison of b and c shows that misorientation is more sensitive and varies more systematically with maximum length than with aspect ratio, and that the differences between sample sets is evidenced more clearly by a comparison with length, or a misorientation per unit length as presented elsewhere.

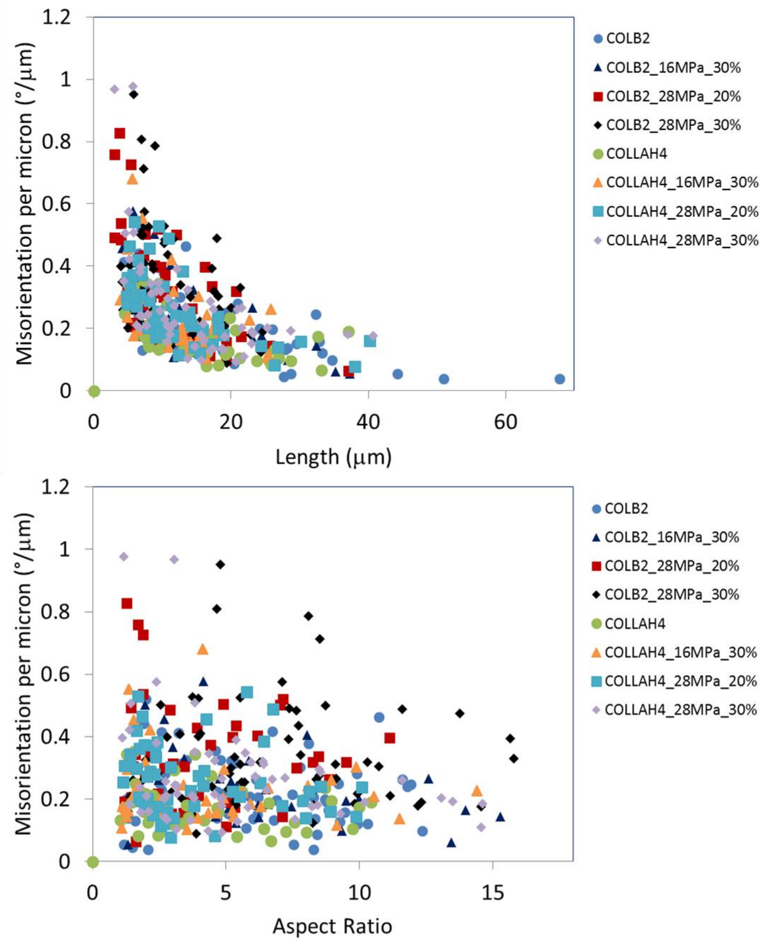

Supplementary Figure 12: A comparison of the relationship of misorientation per micron (amount of distortion) in a microlite as a function of length (top) and aspect ratio (below). This demonstrates that there is a more systematic relationship between distortion and length, with smaller crystals being more distorted.

Supplementary References

1. Kendrick, J. E. *et al.* Tracking the permeable porous network during strain-dependent magmatic flow. *Journal of Volcanology and Geothermal Research* **260**, 117-126 (2013).
