# Supplementary material for: Crystal plasticity as an indicator of the viscous-brittle transition in magmas
Source: Nat Commun. 2017 Dec 4;8:1926. doi: 10.1038/s41467-017-01931-4 (PMC5715024; doi:10.1038/s41467-017-01931-4)
Supplement: Supplementary file 3 — Description of Additional Supplementary Files [file 41467_2017_1931_MOESM3_ESM.pdf]

Description of Additional Supplementary Files

File Name: Supplementary Data 1
Description: An index for the data privided in Supplementary Datasets 2-11, including a description of deformation conditions.

File Name: Supplementary Data 2
Description: EBSD data for sample COLB2 starting material, including: All-Euler Maps of the areas analysed, with microlites numbered as per the order of analysis; details of all the microlites measured including physical properties and; misorientation profiles of all crystals analysed.

File Name: Supplementary Data 3
Description: EBSD data for sample COLB2 deformed at 16 MPa stress and 30 % strain, including: All-Euler Maps of the areas analysed, with microlites numbered as per the order of analysis; details of all the microlites measured including physical properties and; misorientation profiles of all crystals analysed.

File Name: Supplementary Data 4
Description: EBSD data for sample COLB2 deformed at 28 MPa stress and 20 % strain, including: All-Euler Maps of the areas analysed, with microlites numbered as per the order of analysis; details of all the microlites measured including physical properties and; misorientation profiles of all crystals analysed.

File Name: Supplementary Data 5
Description: EBSD data for sample COLB2 deformed at 28 MPa stress and 30 % strain, including: All-Euler Maps of the areas analysed, with microlites numbered as per the order of analysis; details of all the microlites measured including physical properties and; misorientation profiles of all crystals analysed.

File Name: Supplementary Data 6
Description: EBSD data for sample COLLAH4 starting material, including: All-Euler Maps of the areas analysed, with microlites numbered as per the order of analysis; details of all the microlites measured including physical properties and; misorientation profiles of all crystals analysed.

File Name: Supplementary Data 7
Description: EBSD data for sample COLLAH4 deformed at 16 MPa stress and 30 % strain, including: All-Euler Maps of the areas analysed, with microlites numbered as per the order of analysis; details of all the microlites measured including physical properties and; misorientation profiles of all crystals analysed.

File Name: Supplementary Data 8
Description: EBSD data for sample COLLAH4 deformed at 28 MPa stress and 20 % strain, including: All-Euler Maps of the areas analysed, with microlites numbered as per the order of analysis; details of all the microlites measured including physical properties and; misorientation profiles of all crystals analysed.

File Name: Supplementary Data 9
Description: EBSD data for sample COLLAH4 deformed at 28 MPa stress and 30 % strain, including: All-Euler Maps of the areas analysed, with microlites numbered as per the order of analysis; details

of all the microlites measured including physical properties and; misorientation profiles of all crystals analysed.

File Name: Supplementary Data 10
Description: EBSD data for the broken microlites across all samples of COLB2, including: details of all the microlites measured including physical properties, and; misorientation profiles of all crystals analysed.

File Name: Supplementary Data 11
Description: EBSD data for the broken microlites across all samples of COLLAH4, including: details of all the microlites measured including physical properties, and; misorientation profiles of all crystals analysed.
